# Ecological footprint in the OECD countries: do energy efficiency and renewable energy matter?

**DOI:** 10.1007/s11356-024-32151-1

**Published:** 2024-01-31

**Authors:** Thomas Abuobeleye Akpanke, Abraham Deka, Huseyin Ozdeser, Mehdi Seraj

**Affiliations:** Economics Department, Near East University, 99138 Nicosia, Cyprus

**Keywords:** Climate action, Sustainable environment, Technological innovation, Non-renewable energy

## Abstract

Ecological footprint (EFP) measures the amount of area, that is land or sea, which is required to absorb the waste generated through human activities or to support the production of resources consumed by populations. EFP index therefore includes six dimensions that are cropland, forestland, carbon, fishing grounds, grazing land, and built-up area. Human activities have impacted the environment, leading to global warming, widespread droughts, and diseases. The present study aims to investigate the role of renewable energy (RE) and energy efficiency on the EFP index. Past researchers have widely used carbon emission (CE) to represent environmental impact, and recent studies have shown that EFP index is a better proxy of environmental degradation. Therefore, the present research differs from past studies in that it compares on how the determinants of environmental degradation affects EFP index and CE. Panel dataset of the OECD countries from 1990 to 2020 is employed. The CS-ARDL, DCCEMG, and AMG techniques, which overcome dynamics, heterogeneity, and cross-sectional dependence, are employed. The main findings depict that RE significantly reduces EFP and CE, while economic growth significantly exacerbates them. Energy efficiency reduces CE, but does not significantly affect EFP. Non-renewable energy and research & development significantly increase CE, while an insignificant positive effect is observed with EFP. This paper shows that factors that significantly influence CE may not always significantly affect the EFP index. Thus, to reduce environmental degradation it is fundamental to understand on how each dimension of EFP is influenced.

## Introduction

The United Nations (UN) is calling for climate action through its Sustainable Development Goal (SDG) 13. Additionally, SDG 11 calls for the attainment of sustainable cities and communities. Climate action and sustainable cities and communities as presented in the UNSDGs can be achieved if all World nations cooperate and shun activities that are harmful to the environment. This also includes shunning the use of energy sources that are more polluting, like fossil fuels and shift toward the use of safe and clean fuels, such as renewable energy (RE). The use of RE has been recommended in various research studies, though it is said to be expensive when compared with non-renewable energy (NRE) (Becker and Fischer 2013). Thus, various research have been done to examine on how RE development is improved (Mukhtarov et al. 2022). According to Balsalobre-Lorente and Leitão (2020), the European Union (EU) nations are the highest carbon-emitting region. Qin et al. (2021) also provides that China is the largest emitting country and India is the third largest emitting nation. Similarly, the Organization for Economic Cooperation and Development (OECD) countries are among countries that greatly cause environmental degradation. Therefore, the present research seeks to further the growing literature body by examining the various ways that can help alleviate environmental degradation in the OECD region. We take ecological footprint (EFP) index and carbon emission (CE) to proxy environmental degradation (ED) in this study; hence, allows a comparative analysis that examines symmetric or asymmetric effects on EFP and CE.

The activities of humans on the environment are the major cause environmental degradation. EFP is used to proxy environmental degradation because it is widely used to examine human activities’ effects on nature (Alvarado et al. 2022). The need to examine environmental degradation among world economies has tremendously increased due to the devastating effects of global warming and greenhouse effect, which has caused major health and drought effects (Deka et al. 2023). There are basically six major factors included in the EFP index: crop land, forest, carbon demand on land, fishing grounds, grazing land, and built-up area (Abid et al. 2022). According to Salim and Rafiq (2012), CE, a factor of EFP, is determined as the major cause of global warming and greenhouse effect. CE is greatly caused by the use of NRE to attain the economic growth goal. Energy use is considered the major factor that affects ED: for example, the use of NRE has been blamed for exacerbating environmental degradation (Akadiri and Adebayo 2022), while the use of renewable energy (RE) has been applauded for reducing CE. Thus, many environmentalists, economists, engineers, and governments have recommended the use of RE to achieve carbon neutrality goal.

The present study acknowledges the postulations of past studies. Previous researches have widely investigated the effect of the determinants of environmental degradation on CE (Banga et al. 2022; Akram et al. 2022). In recent years, EFP has been adopted as a better proxy of environmental degradation (Bashir et al. 2023). Using the EFP index to proxy environmental degradation is essential because EFP is calculated by including the six dimensions, representing the area responsible for harnessing waste produced by human activities or support the production of resources that they consume. However, this may generalize the relationship since the factors of EFP mentioned earlier are different and may be affected by different factors or same factors in different ways. Therefore, this research is essential in adding to the growing literature body in that in provides an analysis that compares the impact on EFP and CE. The present research helps to ascertain if EFP and CE receives symmetric or asymmetric effects from the various determinants of environmental degradation. Thus, this research is determined to ascertain and assess the role of RE, energy efficiency, research & development (R&D), economic growth, and NRE on the EFP and CE of the OECD countries for the period that ranges from 1990 to 2020. The research specifies two separate models, one with EFP and the other with CE as the proxy of environmental impact (EI). The cross-sectionally augmented autoregressive distributive lag (CS-ARDL), dynamic common correlated effects mean group (DCCEMG), and augmented mean group (AMG) methods that provides reliable results in the presence of heterogeneity, dynamics, and cross-sectional dependence (CD) are employed.

The present research has the following structure: the second section is the literature review section. In that section, the findings of various empirical studies are presented, analyzed, and criticized accordingly. The third section gives an overview on the major theories of ED, which is the basis of this research. The fourth section is the methodology and data section that presents the present research model, the data used, and the method employed in analyzing the relationship specified in the present research. The fifth section presents the findings of the present research and the sixth section discusses the results of the current study with past studies results. Lastly, the study conclusions are given, citing the major policies derived from the study.

## Literature review

### Energy (RE and NRE) and environmental degradation

Literature presents the results of various studies that have been employed to examine the link between the use of energy and ED. Some studies have examined how total energy use or supply impacts ED, while the other studies further examined on how the different energy sources (that is, NRE and RE) impacts ED. Moreover, various proxies have been employed to represent ED. For example, literature provides an extensive range of studies that have employed CE to represent ED. CE remains the main contributor to ED; hence, global warming issues. Recent researches have shifted to the use of EFP, a better proxy if ED.

Firstly, the influence of energy use on EFP has been examined and a positive strong effect has been found to exist (see in Amer et al. 2022; Ali et al. 2022a, 2022b). Additionally, the research of Abbas et al. (2021) ascertains that energy use worsens EFP of Pakistan. Other World regions have been observed to exhibit a strong effect of energy use on EFP that is positive, for example in the GCC countries, in a study of Amer et al. (2022). Why do energy use worsens ED through raising the EFP and CE? Deka et al. (2023) on the examination of the influence of energy use on CE postulates that NRE inclusion in the total energy mix is the core cause of a positive effect between these factors. This notion is supported by the various researches that provide that NRE cause CE to rise in various regions (see for example in the research of Ansari 2022). While the utilization of NRE worsens the degradation of surroundings through either raising EFP or CE, RE is friendly to the surroundings and lowers ED (Abid et al. 2022). Therefore, countries that use more of NRE than RE will exhibit a significant positive effect of energy use on ED, while those nations with relatively lower NRE use in the total energy mix might likely exhibit a negative effect. The other variable that significantly reduces ED, alongside RE, is natural resources. The postulations presented in the research of Abid et al. (2022) alludes that natural resources are key in abating the negative effects presented on the environment.

Secondly, as mentioned above, RE is essential in lowering ED and a good number of papers have been ascertained this link. For example, the research of Bhat (2018), Mathiesen et al. (2011), and Abbas et al. (2021) depicts that the emission of carbon-dioxide (CO_2_) is greatly lowered through the use of RE. Therefore, the findings presented in the researches done in the past depicts the significance of RE in fostering the quality of the surroundings. Akadiri and Adebayo (2022) and Banga et al. (2022) also support the findings that depicts that RE improves the quality of surroundings. RE plays a fundamental role in unlocking the dilemma on the goals meant to enhance quality environment with those seeking to enhance economic growth. This is because energy use worsens ED and improves economic growth; hence, uncertainty on the target goal to pursue arises. RE is observed in the empirical study results to improve economic growth (Kadir et al. 2023, and many others). Therefore, if RE lowers ED and enhances economic growth, then using it is the best way forward to attain environmental sustainability.

However, literature also shows that few researches done in other regions show RE raises CE (see for example, Anser et al. 2021). The study of Adedoyin et al. (2020) also supports this notion by providing that it is the generation of RE that worsens ED through raising CE. Other studies have also shown that the link between CE and RE is not significant. Thus, in as much as many researches show that RE is vital lessening the effects presented on the surroundings, few other researches depict that RE actually worsens ED on the one hand and a no significant link is observed on the other side. The generation of RE of course may worsen ED because of the harmful ways employed in the process. Becker and Fischer (2013) have provided that RE has high costs in the short term, in comparison to NRE, though the long-term costs of NRE are always higher. By this way, we can understand how RE may foster ED or present an insignificant effect because of the high prices.

### Energy efficiency, technological innovations, economic growth, and environmental degradation

The postulations of past empirical studies presented in the section above depict the significance of natural resources and more specifically RE in lessening ED. However, energy efficiency too is fundamental in lessening ED. For example, in Akram et al. (2022), a significant negative effect of energy efficiency on ED is provided. These postulations provide the significance of using energy in a way that avoids wastage in order to foster environmental quality (Deka et al. 2023). For instance, Zakari et al. (2022) give that the levels of CE are reduced by energy efficiency. This is also supported by the postulations of Ponce and Khan (2021). Besides reducing ED, energy efficiency I had also applauded for enhancing economic development (Razzaq et al. 2021). The positive impact of energy efficiency on the attainment of high economic development is also presented in the study of Sohag et al. (2021). The rationale behind the negative effects of energy efficiency on the ED is because high-energy efficiency is associated with the use of limited energy amount to produce more output (Li and Colombier 2009). Few other researches, such as Mahapatra and Irfan (2021), postulates by presenting the inconsistencies of energy efficiency effects on CE.

Recent studies have also presented the importance of technological innovations in reducing the impact imposed by human activities on the environment (Bashir et al. 2023; Ali et al. 2023; Appiah et al. 2023; Ahmad et al. 2020). The aforementioned studies depicts that EFP and CE are reduced through improvements in technological innovations. The proxies of technological innovations that has been used in recent studies include patents and R&D. Due to this reason, the present research also employs R&D as a proxy of technological innovation tom examine its effects of EFP and CE. Other recent studies have also shown that climate technology, environmental regulation, and energy transition enhances the quality of the environment (Ahmad et al. 2023a). The negative effect of environmental regulation is also supported in the research of Meng et al. (2022). Ahmad et al. (2023b) also supports the importance of energy transition in mitigating CE, by providing that green energy transition is key in lowering CE in the E7 countries. Therefore, apart from energy efficiency and RE, various other factors significantly improve environmental quality, and these should be capitalized on by countries to attain the UNSDGs 11 and 13.

In the section above , we have presented NRE as the key driver to ED. Empirical findings presented in the literature have also provided economic growth to be significantly linked with ED, positively. The research of Abid et al. (2022) shows that GDP worsens EFP. The postulations of Ali et al. (2022a, 2022b) who provides that economic development strongly worsens ecological pressure supports the notion presented above. Other studies have also shown that GDP worsens CE, hence ED is exacerbated (Asif et al. 2021; Ansari 2022). The research of Bouyghrissi et al. (2021) also supports that economic growth worsens CE. The strong positive effect of economic growth on ED is traced from the utilization of more polluting energy sources, like NRE and the engagement in harmful activities to improve economic development. Therefore, it is fundamental to ensure that clean energy sources are used to enhance economic development. Attempting to improve economic development with more polluting energy sources is detrimental to the environment. Economic development is essential, so is the clean environment. Thus, both goals must be prioritized and this is attained through the use of RE.

### Research gap and contribution

The empirical literature review presented above gives a wide range of research outcomes on the factors influencing EFP. The findings presented above also depicts that a good number of researches have also employed carbon footprint, a factor of EFP, which represents ED. The present research shows the existence of mixed findings on how various factors impacts EFP and CE, which shows that there is, still, need for more work to be done to ascertain the link between these factors and the explanatory factors. We also observe that EFP index and CE are used to proxy environmental degradation in different studies. The present research differs from past studies because it simultaneously presents two models of environmental degradation, one with EFP and another with CE as the dependent variable. Thus, the present research seeks to compare and contrast on how these factors of environmental degradation are affected by its various determinants. Asymmetric effects on EFP and CE are determined; hence, better policies are presented to attain sustainable cities and communities, and climate action (the UNSDGs 11 and 13, respectively). Most importantly, the research seeks to investigate if energy efficiency and RE significantly reduces both EFP and CE in the OECD countries.

## Theoretical background

There are many theories of environmental that have been supported by empirical studies in recent years and the environmental Kuznets curve (EKC) is among those (Khan et al. 2019; Majeed and Luni 2019; Chang 2010; Jardon et al. 2017). The EKC theory is pioneered through a series of studies; for instance, Shafik (1994), Selden and Song (1994), and Grossman and Krueger (1995), and it provides that ED and GDP growth are linked by way of an inverted U-shape. This implies that low-income level countries that are still growing exhibit a positive association between their growth and ED, while high-income countries will have a negative association between GDP growth and ED. This often results from the fact that the major goal of low-income countries is to promote the economy; hence, may end up doing so at the expense of the environment. High-income countries tend to shift their targets from strictly economic growth oriented to ensuring the attainment of sustainability on the environment. The negative link between economic growth and ED after the turning point can also be explained by the energy transition of nations, from using NRE to RE. Thus, the use of RE allows nations to continue attaining high growth rate alongside lower levels of ED. The EKC hypothesis is named after the Kuznets (1955) who ascertained that the level of income and income inequalities have a significant inverted U-shaped connection. Various other empirical studies have supported the credibility of the EKC theory, by ascertaining that indeed economic growth and ED are linked (Wahab et al. 2020; Safi et al. 2021; Rahman and Mamun 2016; Hussain et al. 2012). On the other hand, we have few other studies that have shown that ED and economic growth are not strongly connected (Cowan et al. 2014; Rahman and Mamun 2016). However, this is not enough to query the credibility of the EKC theory, thus this paper considers economic growth as a core input of ED in the model.

Moreover, the environmental transition theory (ETT) is by far one of the most essential theories of ED that have been widely used in literature. The ETT hypothesis postulates that economic development and environmental quality are dynamically connected. Economic development is observed to be exhibit high levels ED in the initial stage and this is known as the brown agenda. The grey agenda is faced in the second stage as cities grow economically. The grey agenda refers to ED that is connected with auto- and industrial-related pollution, while the brown agenda is connected with water, sewage and sanitation problems. In the third phase, societies faces problems related to the green agenda, that is, the emission of greenhouse gases and depletion of ozone. The ETT hypothesis is generally considered a theory of urbanization, alongside the compact city theory (CCT) and the environmental modernization theory (EMT) (Shah et al. 2020; Poumanyvong and Kaneko 2010).

## Model, data, and methodology

### Model specification

The model specification followed in this research is based on the theories of environmental degradation presented above, that is, the EKC and ETT theories. The EKC (Shafik 1994; Ma et al. 2021; Grossman and Krueger 1995) hypothesis depicts the significance of economic growth in affecting ED, and presents that economic growth and environmental stress are linked in an inverted U-shaped association. Therefore, we consider economic growth as the first and main factor influencing EFP, and specify it in the model. The ETT also presents energy as the key indicator responsible for influencing economic growth. Energy is generally divided into two main branches, that is, RE and NRE; hence, these factors are specified in the present model as the major inputs of EFP. In addition to economic growth, RE and NRE, energy efficiency, and R&D are also specified as the factors affecting EFP. Energy efficiency has been identified as one of the main factors alongside RE, which helps in attaining environmental quality (Zakari et al. 2022; Deka et al. 2023). Therefore, it is vital to include energy efficiency as one of the explanatory series in the model. R&D is employed to proxy technological innovation, a key factor in attaining environmental quality as per the results of past studies (Ali et al. 2023; Bashir et al. 2023). The followed in this study is specified in Equation 1, in functional form.

1 $$EI=f\left( EG, RE, NRE, EE,R\&D\right)$$

In Eq. 1, we show that *EI* is environmental impact (represented by *EFP* and *CE* in this research), which is the explained series. *EG* is economic growth; *EE* depicts the energy efficiency; *NRE* is the usage of fossil fuel power; while *RE* is the utilization of renewable energy power; and R&D development is used to proxy technological innovations.

The present research seeks to compare and contrast on the factors affecting environmental degradation by employing EFP as the dependent variable in one model and CE in another model. Equation 2 presents the first model that has EFP as the dependent variable, while Eq. 3 presents the second model that has CE as the dependent variable.

**Model 1:**

2 $${EFP}_t={\beta}_0+{\beta}_1{RE}_t+{\beta}_2{NRE}_t+{\beta}_3{EE}_t+{\beta}_4R\&{D}_t+{\beta}_5{EG}_t+\mu$$

**Model 2:**

3 $${lgCE}_t={\beta}_0+{\beta}_1{RE}_t+{\beta}_2{NRE}_t+{\beta}_3{EE}_t+{\beta}_4R\&{D}_t+{\beta}_5{EG}_t+\mu$$

In Eq. 2, *EFP* represents ecological footprint. In Eq. 3*CE* represents the emission of CO_2_, while *lg* is the log of a variable. In Eqs. 2 and 3, *β*_0_ is the constant of the statistical model; *β*_1_ to *β*_5_ are the coefficient parameters of the regressors in the models, while *μ* is the white noise error term.

### Data

The data set used in this research is of the OECD nations from year 1990 to the year 2020. The data of CE, R&D, RE, EE, economic growth, and NRE is collected from the World Bank databases, thus World Bank Development Indicators (WDI) are used. EFP data is collected from the Global Network Footprint (GFN) databases. EFP is used to represent environmental degradation in the first model. It is an index calculated by considering six factors that supports human activities or harness the waste produced, that is, cropland, grazing land, carbon, built-up area, fishing grounds, and forestland (Global Network Footprint 2023). EFP in the present research is measured as the total consumption per capita. CE is the emission of CO2 into the air due to the utilization of fossil fuels sources. CE is employed as the dependent variable in Model 2. Economic growth is the change in the GDP of a country every year, whereby a positive value depicts economic growth and a negative value depicts economic decline (Mankiw 2010). RE is the usage of those energy sources that are friendly to the surroundings and can be recycled for use once more. The data of RE collected in the present research is measured as a percentage of total supply of energy (World Bank 2023). Energy efficiency refers to the number of outputs that is produced per each unit of energy employed. When a single unit of energy produces more output, then the energy is said to be efficient. Energy efficiency in this research is calculated as the level of GDP per each unit of energy. Therefore, it is obtained by dividing GDP per capita with total energy supply per capita. NRE are those energy sources which cannot be recycled for use once more, meaning that they get depleted after use. Fossil fuel energy is employed to represent NRE in this research and is measured as a percentage of total supply of energy (World Bank 2023). Table 1 presents a summary on the features of all factors employed in this research.

**Table 1:** Variable summary

Name of variable	Abbreviation	Type of variable	Measurement	Source
Ecological footprint	EFP	Dependent	Consumption per capita	GFN
Carbon emissions	CE	Dependent	Metric tons per capita of CO2 emissions	WDI
Research & Development	R&D	Independent	Expenditure (% of GDP)	WDI
Renewable energy use	RE	Independent	% of total energy use	WDI
Energy efficiency	EE	Independent	GDP per unit of energy (per capita)	WDI
Economic growth	EG	Independent	GDP growth (%)	WDI
Non-renewable energy	NRE	Independent	% of total energy use	WDI

The descriptive statistics of the variables employed in this research are presented in Table 2. This is key in showing the variable characteristics and their measure of dispersion and variance.

**Table 2:** Descriptive statistics

*Variable*	*Obs*	*Mean*	*Std. Dev.*	*Min*	*Max*
*EFP*	1023	5.528704	2.371182	.7686697	17.28278
*RE*	1023	17.46378	13.9111	.44	61.37
*NRE*	1023	73.69266	17.68967	13.05622	98.52626
*EE*	1023	7.25768	4.315714	.2580595	28.99569
*EG*	1023	2.558265	3.297053	− 14.83861	13.05001
*R&D*	1023	1.616994	1.056021	.0091978	5.43562
*CE*	1023	357,660.7	905,037.1	2871.2	5,775,807

### Methodology

Just like any other methodologies followed by past researches, it is vital to begin by running the preliminary tests on the panel variables and the research model specified in order to identify the suitable methodologies to use. The present research begins by testing CD in each variable specified, because panel data usually exhibit CD due to the interrelatedness and trade among nations (Pesaran 2004). Testing for CD is vital in identifying the most appropriate unit root techniques to use. Indicators with significant CD can be checked for stationarity with the second-generation (SG) tools, which have the capacity of overcoming CD and presence robust outcomes. Moreover, since panel dataset in the present research has significant CD, the SG techniques, the CIPS, and the CADF tools are utilized to ascertain the existence of unit root (Im et al. 2003; Pesaran 2007). Checking the existence of unit root is significant in determining the most appropriate method to use for data analysis.

On the preliminary tests of the present research models, the variance inflation factor (VIF) method is used to test the existence of multi-collinearity among independent variables. A VIF value greater than 10 shows that the independent variable has significant multi-collinearity, while a VIF less than 10 shows no problems of multi-collinearity. Moreover, the panel variables employed have different integration orders, some have one order, while others have zero orders; hence, the Westerlund (2007) error correction model (ECM) is used to check cointegration in the research models. It is also essential to ascertain cointegration in the models in order to employ a method that presents long-run estimates in the case of a significant cointegration in the model. Heterogeneity is also tested in the models by employing the Pesaran and Yamagata (2008) slope heterogeneity tests. Models that have significant slope heterogeneity as per the results of the delta and delta-adjusted tests can be analyzed by employing SG methods that overcomes this problem. We also test the presence of weak CD in the research model by employing there tests, that is, the Frees (1995, 2004), Friedman (1937), and the Pesaran (2015) scaled Lagrange multiplier (LM) methods. Models that have significantly weak CD can be analyzed by SG methods that overcome CD.

In this research, because slope heterogeneity, and weak CD are present, we use the CS-ARDL method to examine the relationship of the two models presented in this study. The CS-ARDL method is the SG method that overcomes CD, heterogeneity, and dynamics and (Chudik and Pesaran 2015). The statistical relationship of the CS-ARDL method for the present research models is given in Eqs. 4 and 5. Equation 4 is the CS-ARDL statistical model for Model 1, while Model 2 is presented in Eq. 5.

4 $$\begin{array}{l}{EFP}_t=\beta_0+\sum\limits_{i=1}^p\beta_{1i}\Delta{EFP}_{t-i}+\sum\limits_{i=1}^q\beta_{2i}\Delta N{RE}_{t-i}\\\;\;\;\;\;\;\;\;\;\;+\sum\limits_{i=1}^q\beta_{3i}\Delta{RE}_{t-i}+\sum\limits_{i=1}^q\beta_{4i}\Delta R\&D_{t-i}+\sum\limits_{i=1}^q\beta_{5i}\Delta{EE}_{t-i}\\\;\;\;\;\;\;\;\;\;\;+\sum\limits_{i=1}^q\beta_{6i}\Delta{EG}_{t-i}+\beta_{7i}{NRE}_{t-1}+\beta_{8i}{RE}_{t-1}+\beta_{9i}R\&D_{t-1}\\\;\;\;\;\;\;\;\;\;\;+\beta_{10i}{EE}_{t-1}+\beta_{11i}{EG}_{t-1}+\beta_{12i}{ECT}_{t-1}+\mu\end{array}$$

5 $$\begin{array}{l}{lgCE}_t=\beta_0+\sum\limits_{i=1}^p\beta_{1i}\Delta{EFP}_{t-i}+\sum\limits_{i=1}^q\beta_{2i}\Delta N{RE}_{t-i}\\\;\;\;\;\;\;\;\;\;\;+\sum\limits_{i=1}^q\beta_{3i}\Delta{RE}_{t-i}+\sum\limits_{i=1}^q\beta_{4i}\Delta R\&D_{t-i}+\sum\limits_{i=1}^q\beta_{5i}\Delta{EE}_{t-i}\\\;\;\;\;\;\;\;\;\;\;+\sum\limits_{i=1}^q\beta_{6i}\Delta{EG}_{t-i}+\beta_{7i}{NRE}_{t-1}+\beta_{8i}{RE}_{t-1}\\\;\;\;\;\;\;\;\;\;\;+\beta_{9i}R\&D_{t-1}+\beta_{10i}{EE}_{t-1}+\beta_{11i}{EG}_{t-1}+\beta_{12i}{ECT}_{t-1}+\mu\end{array}$$

In the statistical representation given in Eqs. 4 and 5, *β*_0_ is the constant term; *β*_1_ to *β*_6_ are the coefficient parameters of the short-run estimates; *β*_7_ to *β*_11_ are the coefficient parameters of the long-run estimates; *β*_12_ is the constant of the error correction term (ECT); *μ* is the error term.

Moreover, the DCCEMG method, of Pesaran (2006) and the AMG method, of Eberhardt and Bond (2009), and Eberhardt and Teal (2010) are used to ensure the results of the CS-ARDL method are robust. The methods overcome CD, heterogeneity, and dynamics, just like the CS-ARDL method.

## Results

This study begins by presenting the findings of the CD tool to ascertain if the panel indicators specified in the present research have CD or not (Pesaran 2004). The findings of CD test presented in Table 3 depicts that the indicators, all indicators specified in this research model, have significant CD. Therefore, the indicators specified in the present research are investigated for the presence of unit root with the SG tools that gives robust results in indicators with CD.

**Table 3:** Pesaran (2004) CD test findings

Variables	CD-test	*P* value
*EFP*	34.41***	0.000
*lgCE*	19.63***	0.000
*NRE*	30.82***	0.000
*RE*	48.57***	0.000
*EG*	68.33***	0.000
*EE*	114.72***	0.000
*R&D*	62.21***	0.000

***, **, and * stands for 1%, 5%, and 10% significant level

The present research uses the CADF and CIPS methods to ascertain the integration orders of the variables specified in the present research. The results of the CADF and CIPS methods are presented in Table 4. The findings presented in Table 4 of the CIPS method when considering 5% significance level, depicts that EFP, economic growth and NRE are stationary at level, depicting that these variables are integrated of order zero. The CIPS method also shows that the log of CE, RE, energy efficiency, and research & development are stationary at first-difference, at 5% significance. Thus, log of CE, RE, energy efficiency, and research & development are integrated of order one. The CADF results concurs with the CIPS results that economic growth is stationary at level and also provides that energy efficiency too is stationary at level. Thus, according to the CADF results economic growth and energy efficiency are integrated of order zero. The CADF results also shows that EFP, log of CE, RE, NRE, and research & development are integrated of order one. Where the findings of the CIPS and CADF methods contradicts, we upheld the results of the CIPS method that gives robust results. This research shows that the indicators used in this study models are integrated orders that are different, that is, some variables are integrated of order zero while others are integrated of order one.

**Table 4:** Unit root results

Variables	CIPS	CADF
*EFP*	− 2.409***	− 1.433
*lgCE*	− 1.880	− 1.426
*RE*	− 1.971	− 1.691
*NRE*	− 2.429***	− 1.986*
*EG*	− 3.470 ***	− 2.600***
*EE*	− 1.806	− 2.124**
*R&D*	− 2.092*	− 1.880
*ΔEFP*		− 3.176***
*ΔlgCE*	− 5.091***	− 2.533***
*ΔRE*	− 5.008***	− 2.724***
*ΔNRE*		− 3.139***
*ΔEE*	− 4.657***	
*ΔR&D*	− 4.651***	− 2.882***

***, **, and * stands for 1%, 5%, and 10% significant level

The VIF method is employed to check the existence of multi-collinearity in the independent variables employed in the research models. The results presented in Table 5 shows that the independent variables of both models have a VIF value that is less than 10, depicting that the models have no significant multi-collinearity.

**Table 5:** VIF results

*Variable*	*VIF*	*1/VIF*	*VIF*	*1/VIF*
	Model 1		Model 2	
*RE*	1.82	0.5496	1.82	0.5496
*NRE*	1.77	0.5634	1.77	0.5634
*EE*	1.34	0.7452	1.34	0.7452
*R&D*	1.31	0.7641	1.31	0.7641
*EG*	1.12	0.8957	1.12	0.8957
*Mean VIF*	1.47		1.47	

It is also essential to examine cointegration in the models specified in the present research. For this purpose, we use the Westerlund (2007) ECM method to check cointegration in the models. In the Model 1, we observe that the Gt results present significant results that shows that the variables in the model have strong long run association (see Table 6). However, the Ga, Pt, and Pa results are insignificant depicting that the model has no significant long run connection. Because of the Gt results, we conclude that the model has a strong long run connection. In the Model 2, we observe that there is no significant long run link because all tests methods of Westerlund (2007) are insignificant.

**Table 6:** Westerlund (2007) ECM cointegration results

*Statistic*	*Value*	*Z value*	*P value*	*Value*	*Z value*	*P value*
	Model 1			Model 2		
*Gt*	− 2.509	1.756**	0.040	− 0.347	10.453	1.000
*Ga*	− 3.701	6.002	1.000	− 0.287	8.556	1.000
*Pt*	− 10.279	0.433	0.668	− 2.922	6.453	1.000
*Pa*	− 4.634	2.454	0.993	− 0.329	5.564	1.000

***, **, and * stands for 1%; 5%, and 10% significant level

It is also essential to investigate the presence of significant heterogeneity in the specified models. We employ the slope heterogeneity test for this purpose (Pesaran and Yamagata 2008). The findings presented in Table 7 depicts that all the two models specified have significant slope heterogeneity. Therefore, the present research employs methodologies of data analysis that are strong in the presence of slope heterogeneity in order to present robust outcomes.

**Table 7:** Slope heterogeneity results

	*Statistic*	*P value*	*Statistic*	*P value*
	Model 1		Model 2	
*∆*	23.934***	0.000	27.263***	0.000
*∆ adj.*	27.201***	0.000	30.984***	0.000

***, **, and * stands for 1%, 5%, and 10% significant level

It is also essential to check the existence of weak CD in the models to determine the correct method of data analysis. The Pesaran (2015) scaled LM, Friedman (1937), and the Frees (1995, 2004) tests are employed for this purpose and their findings are presented in Table 8. The results in Table 8 depict that both models have significant weak CD; hence, methods that overcomes weak CD should be employed to analyze the relationship specified in the present research.

**Table 8:** Weak CD test

*Method*	*Statistic*	*P value*	*Statistic*	*P value*
	Model 1		Model 2	
Pesaran (2015) Scaled LM	27.290***	0.000	2.700***	0.0069
Friedman (1937)	237.973***	0.000	52.014**	0.0141
Frees (1995, 2004)	6.881***	0.000	5.415***	0.000

***, **, and * stands for 1%, 5%, and 10% significant level

The SG methods, that is the CS-ARDL, DCCEMG, and AMG methods, are used to analyze the relationships specified in the Model 1 and 2 of this study. The data analysis methods used are the SG methods that overcomes CD, dynamics, and heterogeneity.

Table 9 gives the results of the CS-ARDL method for Model 1 and 2. The CS-ARDL results depicts that RE reduces EFP, economic growth increases EFP in the OECD countries. The long as well as the short run estimation results concurs that RE reduces EFP and that economic growth worsens EFP. The findings show that an increase in RE by one unit is linked with a decrease in EFP by 0.0498 and 0.06 units, in the short and long term respectively. Therefore, RE is fundamental in improving the quality of the environment through lowering EFP. Moreover, an increase in economic growth is linked with a rise in EFP by 0.0334 and 0.0395 units in the short and long term respectively. This shows that high GDP growth in the OECD countries worsens environmental degradation. NRE, R&D and energy efficiency are observed to present an insignificant effect on EFP in the OECD countries. The long and short run estimations depicts that these indicators does not significantly affect EFP. NRE and energy efficiency have positive effect, both in the short and long run, depicting that these factors can raise EFP, while R&D has a negative coefficient depicting that it may lower EFP.

**Table 9:** CS-ARDL results

	*Coefficient*	*t-Statistic*	*P value*	*Coefficient*	*t-Statistic*	*P value*
	Model 1			Model 2		
*Short run estimations*						
*lgCE(-1)*				0.3028	7.51***	0.000
*EFP(-1)*	0.1139	2.06**	0.039			
*RE*	− 0.0498	− 2.90***	0.004	− 0.0172	− 5.72***	0.000
*NRE*	0.0126	0.68	0.498	0.0063	3.09***	0.002
*EG*	0.0334	3.27***	0.001	0.0043	5.52***	0.000
*EE*	0.0711	1.39	0.164	− 0.0218	− 2.77***	0.006
*R&D*	− 0.2310	− 1.26	0.208	0.0496	1.63	0.103
*ECT(*− *1)*	− 0.8861	− 16.04***	0.000	− 0.6971	17.29***	0.000
*Long run estimations*						
*EE*	0.0549	0.98	0.326	− 0.0359	− 2.60***	0.009
*EG*	0.0395	3.15***	0.002	0.0072	5.44***	0.000
*NRE*	0.0089	0.39	0.699	0.0059	1.74*	0.081
*R&D*	− 0.0665	− 0.28	0.778	0.0923	1.67*	0.094
*RE*	− 0.0611	− 2.76***	0.006	− 0.0317	− 4.43***	0.000

***, **, and * stands for 1%, 5%, and 10% significant level

In the Model 2, the CS-ARDL results depict that EE and RE significantly reduces CE, according to the short and long run estimations. The short and long run findings shows that raising RE by 1% is associated with an increase in CE by 0.017% and 0.032%, respectively. Moreover, raising energy efficiency by 1% is related with a rise in CE by 0.022% and 0.036%, as per the short and long run estimations respectively. Therefore, RE and energy efficiency are key factors leading to a reduction of CE in the OECD. Additionally, the CS-ARDL findings depicts that NRE, R&D and economic growth worsens CE in the OECD countries. However, the positive effect of R&D on CE is only significant in the long run, than in the short run. The short and long run estimation findings present that raising NRE by 1% is connected with a 0.006% and 0.0059%, respectively. Furthermore, raising economic growth by 1%, in the short and long run, leads to an increase in CE by 0.004% and 0.007%, respectively. Moreover, a 1% increase in R&D is linked with an increase in CE by 0.09% in the long run. In the short run, R&D does not significantly affect CE, though the coefficient is positive.

To verify the results of the CS-ARDL method, we use the AMG and DCCEMG methods. The long run results of the AMG and DCCEMG methods for Model 1 and 2 are presented in Table 10.

**Table 10:** AMG and DCCEMG results

	*Coefficient*	*t-Statistic*	*P value*	*Coefficient*	*t-Statistic*	*P value*
	Model 1			Model 2		
*DCCEMG method*						
*lgCE(*− *1)*				− 0.6836	− 17.03***	0.000
*EFP(*− *1)*	− 0.8946	− 14.23***	0.000			
*RE*	− 0.0412	− 2.05**	0.040	− 0.0167	− 5.37***	0.000
*NRE*	0.0127	0.64	0.525	0.0062	3.06***	0.002
*EG*	0.0386	4.00***	0.000	0.0041	5.36***	0.000
*EE*	0.0755	1.46	0.145	− 0.0197	− 2.77***	0.006
*R&D*	− 0.1576	− 0.82	0.410	0.0572	1.66*	0.096
*AMG method*						
*RE*	− 0.0309	− 2.05**	0.040	− 0.0223	5.63***	0.000
*NRE*	0.0130	1.05	0.295	0.0098	4.45***	0.000
*EG*	0.0213	4.87***	0.000	0.0035	4.57***	0.000
*EE*	0.0325	1.73*	0.084	− 0.0089	1.75*	0.080
*R&D*	0.0012	0.01	0.993	0.0088	0.34	0.734

***, **, and * stands for 1%, 5%, and 10% significant level

The DCCEMG method results supports the findings of the CS-ARDL method by presenting that RE significantly reduces EFP, while economic growth significantly increases EFP in in the OECD countries. Raising RE by one unit causes EFP in the OECD countries to drop by 0.04 units. Moreover, raising economic growth by one unit is connected with a rise in EFP by 0.039 units. This shows that RE is key in reducing EFP, while economic exacerbates EFP in this region. Similar to the CS-ARDL results, the DCCEMG results shows that NRE, energy efficiency, and R&D does not significantly affect EFP. Their coefficient values are positive depicting that they can raise EFP in this region.

The AMG results also supports the results of the CS-ARDL and DCCEMG methods on the significant negative influence of RE and positive effect of economic growth on EFP. The results of the method also support the insignificant effect of NRE and energy efficiency on EFP, with positive coefficient values too. However, the AMG method shows that R&D has significant positive effect on EFP. This is not in line with the CS-ARDL and DCCEMG results that presents insignificant link between R&D and EFP. The results shows that raising RE by one unit is linked with a drop in EFP by 0.03 units, while raising economic growth and R&D by one unit is linked with a rise in EFP by 0.02 and 0.0012 units, respectively.

The DCCEMG and the AMG supports the findings of the CS-ARDL method in Model 2 by presenting that RE and energy efficiency reduces CE, while NRE, R&D, and economic growth increases CE in the OECD countries, though the AMG method shows that the effect of R&D is insignificant but positive. An increase in RE by one percent is connected with a decrease in CE by 0.017% and 0.022%, according to the DCCEMG and AMG results respectively. Moreover, raising energy efficiency by one percent is linked with a decrease in CE by 0.02% and 0.009%, according to the DCCEMG and AMG results respectively. The outcomes of the DCCEMG and AMG methods also shows that raising NRE by 1% results in a simultaneous increase in CE by 0.006% and 0.0098%, respectively. Additionally, raising economic growth by 1% is connected with a rise in CE by 0.004% and 0.0035%, according to the results of the DCCEMG and AMG findings respectively. The DCCEMG method also depicts that raising R&D by one percent is related with a rise in CE by 0.057% in the long run. Therefore, R&D, NRE and economic growth are the key factors that exacerbate CE in the OECD. To curb CE in this region, RE use and energy efficiency must be improved.

## Discussion

The findings presented in this research are essential in ascertaining the formulating of proper policies in attaining environmental quality. The present research shows a comparative analysis on the factors that affect EFP, which measures environmental degradation and CE a major component of EFP. This research also uses the three main methods of analyzing the data, that is, CS-ARDL, DCCEMG, and AMG, which presents robust findings regardless of heterogeneity, dynamics, and CD issues. Thus, the findings presented in the present research are essential in ascertaining if RE, energy efficiency, R&D, NRE, and economic growth impacts EFP and CE in the same way.

Firstly, it is observed in the findings that economic growth is significant in promoting EFP and CE in the OECD countries. The results of the CS-ARDL, AMG, and DCCEMG concurs that economic growth contributes to the harming of the environment through exacerbating EFP and the emissions of carbon. The present research findings are related to the findings given by previous researches which shows that economic growth of a country harms the environment (Ali et al. 2022a, 2022b; Akadiri and Adebayo 2022; Ben Mbarek et al. 2018; Bouyghrissi et al. 2021). Therefore, the ways of promoting economic development that do not harm the environment must be adopted. R&D too is observed to raise CE in the OECD countries at 10% level of significant, while its effect on EFP is positive by insignificant. The positive effect of R&D on CE and EFP in the present research is not supported by the findings of past researches that shows that technological innovations improves the cleanliness of the environment (Ali et al. 2022a; Bashir et al. 2023; Appiah et al. 2023; Ahmad et al. 2020). The differences on the present results and those of past studies maybe due to use of different proxies of technological innovations.

The results of the CS-ARDL, AMG, and DCCEMG concurs that NRE gives a positive significant effect on CE, and an insignificant positive effect on EFP. This depicts that the use of NRE in the OECD countries is not favorable to the environment. The postulations presented in this research which depicts that NRE promotes CE, is supported by the postulations which are presented in the researches done in the past (see for instance, Deka et al. 2023; Balsalobre-Lorente and Leitão 2020; Banga et al. 2022; Ansari 2022, among other researches). The findings of past studies together with the findings presented in the current research depicts that NRE should be dropped because of its harmful effects on the environment. It is therefore essential for governments to recommend the shift from using NRE in their nations to using sources of energy that are safe and clean.

The present research outcomes further show the importance of RE and energy efficiency in improving environment quality in the OECD countries. The CS-ARDL, AMG, and DCCEMG findings depicts that improving the use of RE in the OECD countries significantly reduces EFP and CE in this region. The negative effect of RE on EFP and CE is supported by various previous researches (Abbas et al. 2021; Akadiri and Adebayo 2022; Mathiesen et al. 2011; Akram et al. 2022, among other studies). This shows that RE is essential and paramount in protecting the environment, through lowering the level of EFP and CE in the OECD countries. Moreover, energy efficiency is also presented as one of the main factors responsible for lowering the level of CE among the OECD countries. The results of the CS-ARDL, AMG, and CCEMG techniques concurs that energy efficiency significantly reduces the level of carbon footprint in the OECD Countries. These findings are supported by the results presented in the previous researches (Deka et al. 2023; Akram et al. 2022). Therefore, every unit of energy used must be ensured to produce more output. Energy waste should be banned, since this causes environmental degradation. This assertion is supported by the postulations of Li and Colombier (2009) who alludes that energy efficiency reduces the use of energy, hence promoting the quality of the environment. However, EFP is insignificantly affected by energy efficiency, according to the CS-ARDL and DCCEMG methods, while the AMG method shows a positive effect that is significant at 10% level.

## Conclusion

This research is vital in furthering the growing literature body on the factors affecting ecological footprint. The present research presents a comparative analysis on the EFP and CE are influenced by various determinants of environmental degradation. This research recommends the utilization of RE because of its significant negative effect on EFP and CE. RE does not only reduce CE, but rather all factors of environmental degradation as represented by the EFP index. The present research also recommends the use of energy in a way that produces more output per each single energy unit employed, avoiding wastage, because of its negative effect on CE. Asymmetric effects of energy efficiency on CE and EFP are observed, since an insignificant effect of the EFP index is observed. Thus, energy efficiency can greatly reduce CE only, than all factors of EFP index. This research also shows that there are no asymmetric effects of economic growth on CE and EFP. Economic growth raises both EFP and CE, depicting that the growth of the economy should not be improved by employing ways that degrades the surroundings. We also show that NRE use should be shunned in the OECD countries because it significantly worsens CE, though EFP is not significantly affected but a negligible positive effect that is not significant is observed. We also recommend the utilization of green R&D in order to reduce EFP in the OECD countries. The current R&D is this region is not sustaining. Future studies can explore on this relationship by employing other proxies of technological innovations, such as patents.

## Data Availability

The data used in this paper is secondary data and was retrieved from the World Bank, https://data.worldbank.org/ and the Global Footprint Network, https://data.footprintnetwork.org/.
